# Novel light-driven functional AgNPs induce cancer death at extra low concentrations

**DOI:** 10.1038/s41598-021-92689-9

**Published:** 2021-06-24

**Authors:** Ulviye Bunyatova, Manel Ben Hammouda, Jennifer Zhang

**Affiliations:** 1 Biomedical Department, Engineering Facility, Baskent University, Ankara, Turkey; 2 Department of Electrical and Computer Engineering, Pratt School of Engineering, Duke University, Durham, NC USA; 3 Department of Dermatology, School of Medicine, Duke University, Durham, NC USA

**Keywords:** Composites, Nanoparticles

## Abstract

The current study is aimed at preparing light-driven novel functional AgNPs- bio-hydrogel and evaluating anticancer potency against human melanoma cells. With an average size of 16–18 nm, the hydrogel nano-silver particle composite (AgNPs@C_MA_O) was synthesized using a soft white LED approach and analyzed by UV–Vis, DLS, FTIR, X-ray, SEM–EDX and TEM techniques. The anticancer activity of the obtained novel functionalized AgNPs@C_MA_O was tested in-vitro in the A375 melanoma cell line. Dose–response analysis showed that AgNPs at 0.01 mg/mL and 0.005 mg/mL doses reduced the viability of A375 cells by 50% at 24 and 48-h time-points, respectively. A375 cells treated with AgNPs@C_MA_O for 24 h at IC50 displayed abnormal morphology such as detachment edges and feet, shrinkage, membrane damage, and the loss of contact with adjacent cells. Our work is the first study showing that non-ionizing radiation mediated biofunctionalized AgNPs have an anti-tumoral effect at such a low concentration of 0.01 mg/mL. Our approach of using harmless wLED increased synergy between soft biopolymer compounds and AgNPs, and enhanced anticancer efficiency of the AgNPs@C_MA_O biohydrogel. Ultimately, the AgNPs accessed through the use of the wLED approach in colloidal syntheses can open new applications and combinatorial advanced cancer treatments and diagnostics.

## Introduction

Metal nanoparticles (NPs) have shown immense potential in medical applications due to their distinctive physio-chemical and biological properties with the right functionalized coating^1–4^. Among the noble metals, silver has attracted significant attention due to its remarkable medicinal value as a crucial antibacterial and anticancer agent ^5,6^. Silver Nanoparticles (AgNPs) have shown antimicrobial properties against various viruses, bacteria, fungi, protozoa and exert anticancer activity towards human cancer cells^7–10^. However, the use of AgNPs in medical fields remains somewhat limited due to their probable cytotoxic effects. In order to reduce the toxicity, AgNPs can be functionally modified ^11^ by biocompatible polymers consisting of polysaccharides, such as cellulose, chitosan, dextran, starch, with a rich functional side. The targeting properties of AgNPs are also governed by chemical interactions between the functional attachments on the surface of NPs and the receptors on cell surfaces. Herein, AgNPs are designed in novel Carboxymethyl chitosan (CMC) and poly(acrylic acid-co-maleic acid)(MA) hybrid hydrogel in the presence of amine-containing surfactant, octadecylamine(ODA) (hereafter denoted as AgNPs@C_MA_O; and hydrogel part hereafter denoted as Hydrogel_C-MA_O).

CMC,a recently developed polysaccharide, is found to be more potent in the treatment of metastasis and solid tumors ^12–14^. Maleic acid “MA” is a carboxylate rich polymer with linear conjugation of the acrylic and MA units are shown to inhibit solid tumor growth and tumor metastasis ^15–16^. The MA unit is susceptible to nucleophilic attack by hydroxyl or amino groups^13^, which is often presented in the structures of the polysaccharides. On the other hand, these hydroxyl groups are susceptible to esterification reactions. It is also known that carboxyl groups are reductive in the presence of light and provide efficient conditions for *in-situ* generations of cationic Ag+ forms to silver NPs (AgNO3 → AgNPs)^17–18^. ODA with primary amino groups can also modify the surface chemistry and make surface more capable to associate with cells ^19–20^.

For our study, we employed an innovative white Led (wLED) synthesis approach to obtain AgNPs in a hydrogel nanocomposite. Based on the literature, nanofabrication of AgNPs using this harmless wLED method has never been attempted before^21^. To expand the wLED approach, we applied quantitative parameters during the in-situ process. Here, we present the results on the synthesis and characterization of AgNPs in novel biohydrogel, and its cytotoxic effects on the human melanoma A375 cell line.

## Results and discussion

### Synthesis method and pathway

**Figure 1:**
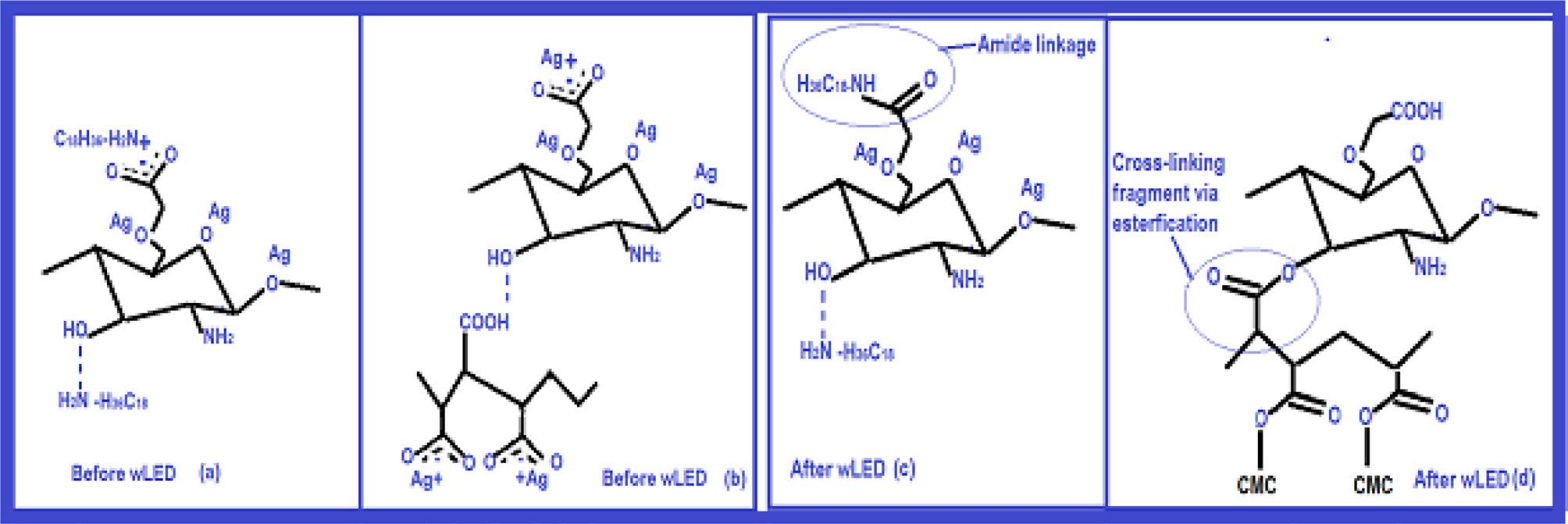
Schematic illustration of syntheses process: **(a)** interaction CMC functional groups with amine groups of ODA via alkyl-NH2 carboxylates complexes and CMC-O- (ether)… +Ag before wLED exposure; **(b)** CMC-OH…HOOC-MA (hydrogen bonding), CMC-COO-… +Ag ions; and MA-COO-… +Ag ions fragments before wLED exposure; **(c)** grafting (amidization) fragment of ODA with CMC carboxyl groups after wLED exposure; **(d)** the esterification or cross-link bonding fragment between CMC and MA macromolecules after LED-treatment.

AgNPs in the C_MA_O mixture were obtained at room temperature using novel wLEd approach. (Schema S1 and Fig. S1, Supporting information (SI)). Colloidal soft sample was placed in a 1 cm quartz cuvette for measurement. All experiments involving irradiation of nanocomposites were performed by employing full spectrum visible light (388–842 nm). The wLED scattering light spectra was recorded using HR 4000 and analyzed by Ocean Software program. The synthesis pathway and fragments of possible physical complexes and chemical bonding taking place in the hybrid composite hydrogel during *in-situ* synthesis and reduction of AgNP are given in Fig. 1 (Fig. S2–S5).

In terms of physical and chemical interactions this novel in situ AgNPs nanofabrication method can be defined by the fragments given in Fig. 1 and lettering as below: CMC − O − (ether)…Ag+ ; CMC − OH (hydroxyl)…Ag+ ; ODA − H_2_N + … − OOC-CMC and (ODA − H_2_N + …OH − CMC (Fig. 1a); MA − COO − … + Ag; CMC − OH…HOOC-MA (hydrogen bonding) (Fig. 1b). wLed irradiation bears the conditions of grafting partially via ODA − H_2_N + … − OOC-CMC (Fig. 1c) and crosslinking/esterification via CMC − OH…HOOC-MA (Fig. 1d) bonds respectively. wLED creates an exquisite setting for reducing ultra stable functionalized AgNPs: the cationic Ag+ easily transforms to the silver NPs (AgNO3 → AgNPs). This carboxyl and amine containing aqueous hybrid matrix not only acted as reducers and stabilizers factor for the in situ generated AgNPs, but also determined the resultant vector of interaction of AgNPs with the surrounding biological environment ^22^.

### Characterization of novel AgNPs@C_MA_O nanohydrogel

**Figure 2:**
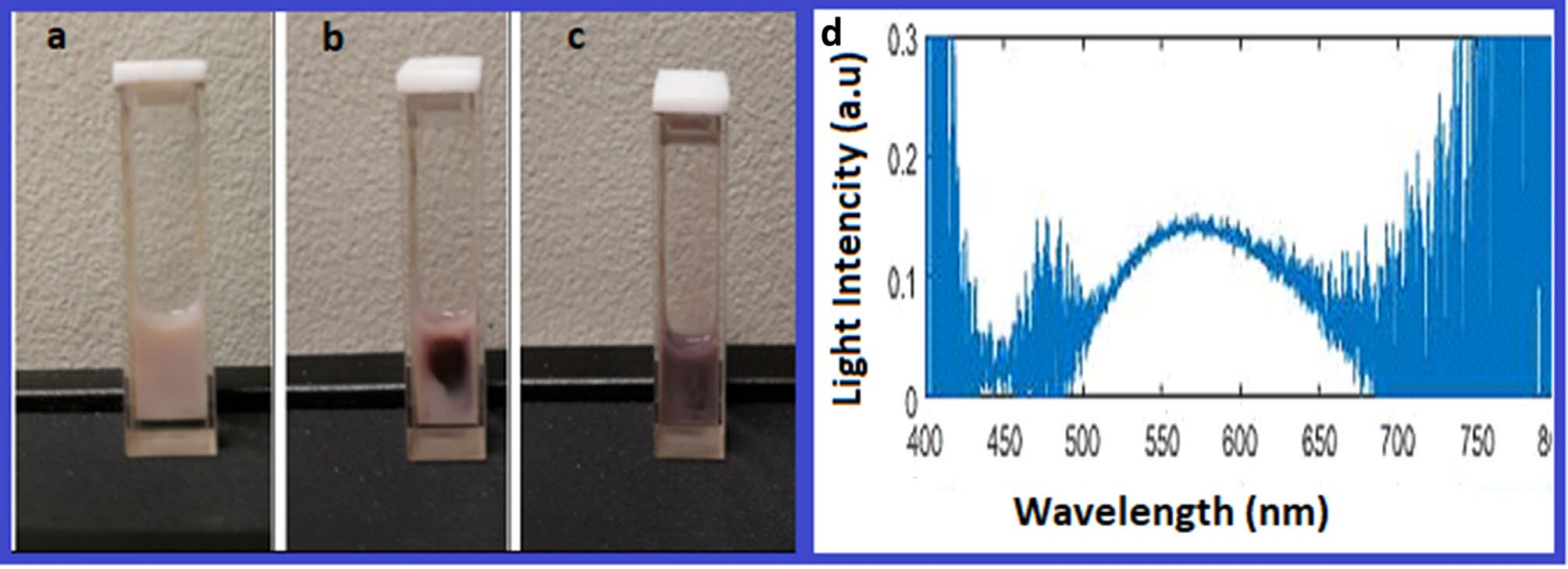
Illustration of soft wLED assisted formation of AgNPs@C_MA_O hydrogel. Nano silver formation in photosensitive hybrid hydrogel matrix was primarily confirmed by the visible color change. The colored ‘dot’ occurs in the center of the spot of wLEd during first minutes of irradiation, then color changes over the cuvette volume due to the irradiation temperature gradient **(a)** before wLEd exposure, **(b)** after 5 min and **(c)** after 25 min of wLED irradiation. **(d)** shows the scattering light spectra under influence of wLED .

As depicted in the Fig. 2a–c, the color changed from pale pink to dark brown after 20–25 min exposure by wLED. To better understand the formation of the AgNPs, we collected quantitative data after two hours exposure and measured the intensity of the scattering light from the sample during this irradiation time.

The obtained results show that the scattering light intensity changes over time and reaches saturation by 20–30 min (Fig. 2d, Fig. S1) indicating that scatterıng light intensity is responsive to the exposure to the wLED source. A previous study demonstrated that any variation in particle size, shape, or dielectric environment will change their scattering, absorption, and extinction responses ^23–24^. Due to particle size sensitivity, we showed the scattering light occurred and changed through in-situ the generation and reduction of the AgNPs. Our data on time-dependent changes in light scattering can be used as an indicator of the in situ production of the AgNPs. We have examined scattering light intensity for 2 h and recorded spectrum data at a 2-min interval. When we analyzed data over time we observed that at the first minutes of radiation the intensity of scattering reach its peak at 566 nm, then the scattering light intensity decreased during 20–30 min. As expected, the peak decreased and became saturated over time indicating the wLED assisting activity to the reduction of AgNPs. The reduction peak decreased and only varied ~ 3%, indicating that AgNPs have good stability inside nanohydrogel matrix (Fig. S1). Therefore, this harmless, simple, one-pot nanofabrication technique at ambient temperature and atmospheric pressure, can be used for formation ultra stable AgNPs. It is notable, that no color change was observed in the matrix suspension without silver ions. On the other hand, as we described above, the reduction of the Ag+ to AgNPs in hydrogel matrix also accompanied by a color change from pale pink to dark brown. The formation of the dark brown color in the reaction mixture also indicated the excitation of Surface Plasmon Resonance (SPR) of AgNPs^25^ . SPR absorption is a unique property of metal nanoparticles and it is arises due to the presence of free electrons in the conduction band.

**Figure 3:**
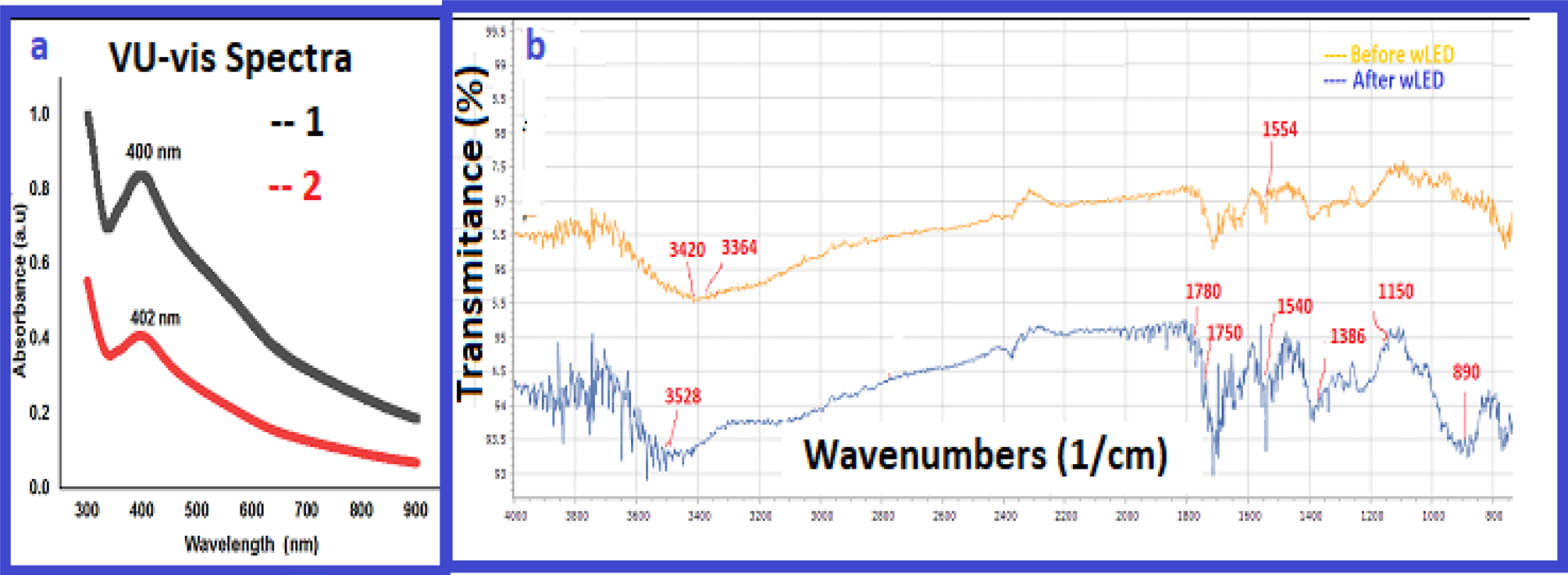
**(a)** UV–vis spectra of the AgNPs@C_MA_O **(a)** red spectra 1; and black spectra 2 is a spectra of a control group of free AgNPs, verifies that AgNPs in the AgNPs@C_MA_O not aggregated, **(b)** FTIR spectra of the AgNPs@C_MA_O before and after wLED treatment.

It is well known that silver nanoparticles absorb light in the visible region of the electromagnetic spectrum (380–450 nm). Therefore, the UV–visible spectroscopy method has been considered as a quick and simple approach to obtain nanoparticles. This preliminary analysis was performed for 0.05% transparent suspension of AgNPs@C_MA_O. The characteristic silver SPR absorption band was observed at 402 nm ^26,27^, indicating a successful formation of AgNPs without aggregation (Fig. 3a).

Next, the FTIR, DLS, EDS and XRD techniques were detected to analyze the functional groups, covalent bonding information, nanoparticles size, surface potential, the elements and crystal structure. C_MA_O and AgNPs@C_MA samples before and after wLED treatment were subjected to the FTIR analysis as shown in SI (Figure S5_1) and Fig. 3b respectively. We can notice a broad peak (SI; Fig. S5_1) around 3600–3200 cm^−1^ which corresponds to O–H groups and N–H stretching vibrations in the spectra hydrogel-MA_O sample. The spectra peak at 1690 cm^−1^ is assigned to the carbonyl groups; symmetrical stretching vibration (COO) absorption peak are observed at 1412 cm^−1^. The carboxylic acid OH stretching at 3364 cm^−1^ in spectra nanocomplex hydrogel before wLED (Fig. 3b) treatment significantly shifted to the higher wavelength region 3528 cm^−1^ after wLED treatment sample due to structural changes from the complexes physical and chemical interactions of CMC–OH groups to form the covalence ester carboxylate linkages with copolymer macromolecules. Absorption bands for the ester carboxylate linkages disappeared, broad stretching bands of pure of CMC –OH groups were shifted from 3420 cm^−1^ to 3536 cm^−1^, intensity of C = O band at 1543 cm^−1^ from hydrogen bonded –COO– group was visibly increased due to loading surfactant ^28^. The characteristic bending deformation band at 1386 cm^−1^ and stretching deformation at 2890 cm^−1^ of the CH_3_ end-linkage group of octadecyl amine appeared only after LED treatment of NC ^28^*.* An absorption stretching band at 1554 cm^−1^ is associated with Ag salts carboxylic acid complexes (–COO- + Ag). This peak disappeared after LED-treatment with the formation of a new strong absorption band at 1540 cm^−1^(vs), which is related to NH deformation^29^ from a secondary amide linkage (1530 ± 30 cm^−1^) (–NH–C=O) as a result of grafting the CMC-COOH and Copolymer-COOH with octadecyl amine surfactant. Two new stretching absorptions were found after LED-treatment related to the − C − O − C − ester group at 1150 and 1160 cm^−1^ from cross-linking the fragments ^28,29^. A new absorption band appeared at 1865 cm^−1^ after LED-treatment related to C=O carbonyl groups stretch of the cross-linking fragments. Tow new stretching bands separated by 30 cm^−1^ were observed at 1750 cm^−1^ and 1780 cm^−1^ –C=O carbonyl groups of co-polymers. Absorption bands at 720 cm^−1^ represent the CH2 rocking vibration band in methylene chains. This chain –(CH2)n– appears as an octadecyl fragment of ODA- structure. The wLEd-induced sharp band at 890 suggests bending vibration of =C–H & =CH2. The above observation can be explained by increasing in situ the interfacial physical interactions such as complexing via hydrogen bonding between amine groups and functional groups of CMCs (e.g. carboxyl and hydroxyl). These findings confirm that wLED light synergistically regulates the reactivity of photoactive functional linkages and silver cations via transferring these fragments to the strong covalent bonds and assisting in the in situ reduction of AgNPs.

**Figure 4:**
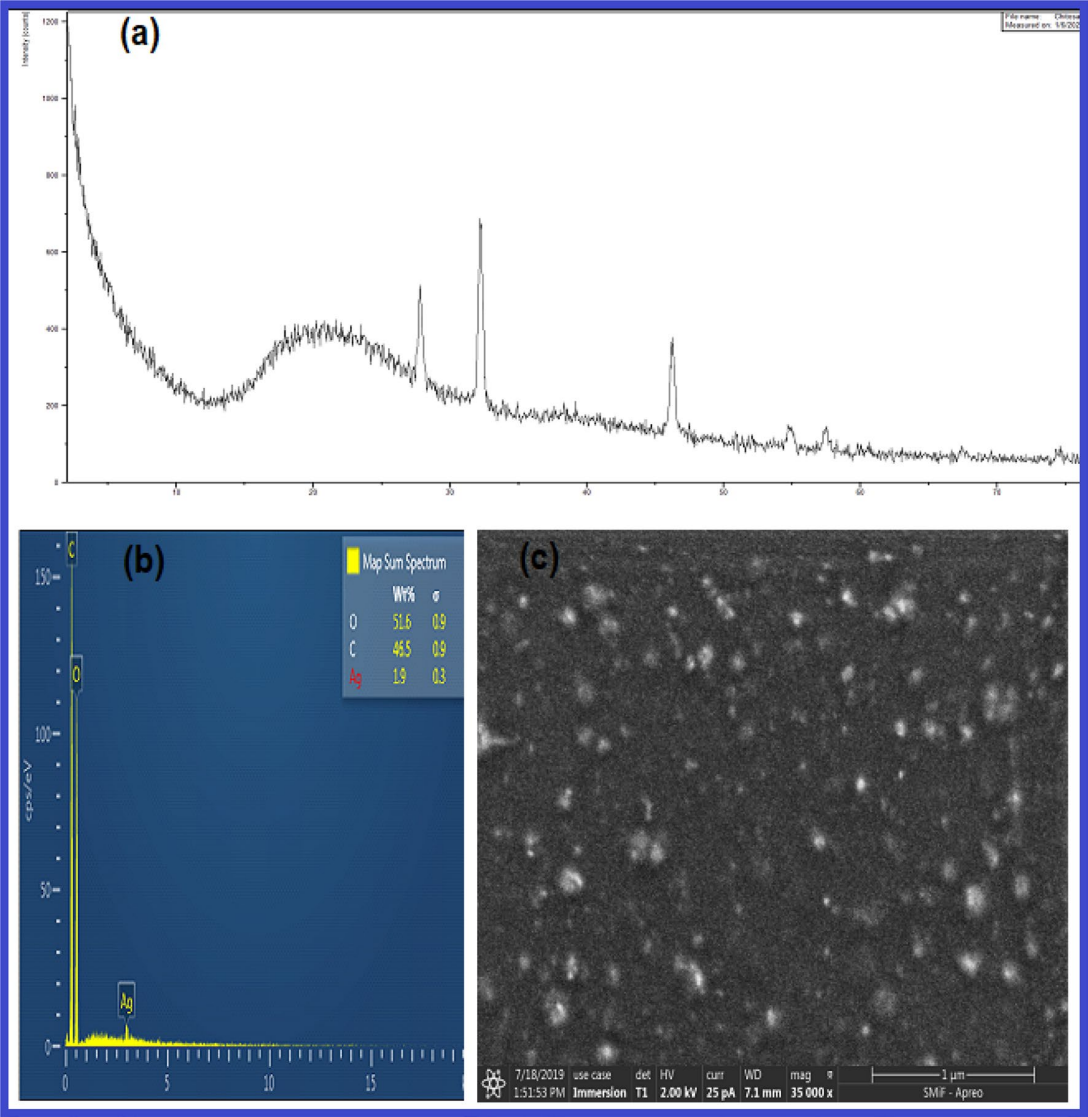
XRD pattern of **(a)** EDS elemental analysis; **(b)** SEM; **(c)** image of the AgNPs@C_MA_O obtained from dryed spincoated film.

The particle size and Zeta potential of the obtained AgNPs@C_MA_O were characterized by dynamic light scattering (DLS). The average size of AgNPs measured from DLS was found to be 24 nm. (Fig. S6). Next, we measured the potential difference between the dispersion medium and the stationary layer of hydrogel attached to the dispersed particle, which is known as Zeta potential. The obtained surface potential value of the AgNPs@C_MA_O was + 18.2 mV (Fig.S7). It is known that the AgNPs tend to aggregate to larger structures during post-synthesis phase. Some researchers theorize that a Zeta potential value outside the range of − 25 mV to + 25 mV prevents NPs against self-aggregation^30^. However, our AgNPs showed better quality and higher degree of stability with unusual antı-cancer potential. To further identify the structure of the AgNPs@C_MA_O nanocomposite, the corresponding XRD and EDS/SEM analyses were carried out. The XRD pattern of AgNPs@C_MA_O (Fig. 4a) exhibited three sharp peaks with 2θ at 27.783°, 32.196° and 46.130 , which corresponded to 111, 200, and 200 hkl values of silver NPs respectively ^31,32^. The relatively weak diffraction peaks at 2θ (54, 83, 57, and 52) attributed to the 111 and 200 hkl values. All these peaks represent the Face centered cubic (FCC) structure of the silver element. The position of all those peaks revealed the presence of AgNPs in the hydrogel structure. The average crystalline size ‘D’ of AgNPs was calculated by Debye–Scherrer formula and was found to be 28 nm. The EDS results show strong oxygen and carbon peaks along relatively weak silver signal at 3 keV (Fig. 4b) ^33^. EDS elemental analysis result confirmed the presence of AgNPs in Hybrid Hydrogel as well as its quantitative mass ratio (10:90).

To further determine the sizes, morphology and dispersion of the AgNPs@C_MA_O the SEM and TEM were employed. The structure and morphology of the AgNPs@C_MA_O and hydrogel C_MA_O were characterized by SEM as shown in Fig. 4c. and SI Fig. S9 respectively.

SEM image (Fig. 4c) reveals that the nanoparticles in the hydrogel matrix are monodispersed and mostly spherical in shape and encapsulated onto hybrid hydrogel, while hydrogel_C_MA_O show uniform porous structure (SI. Fig. S9) .

**Figure 5:**
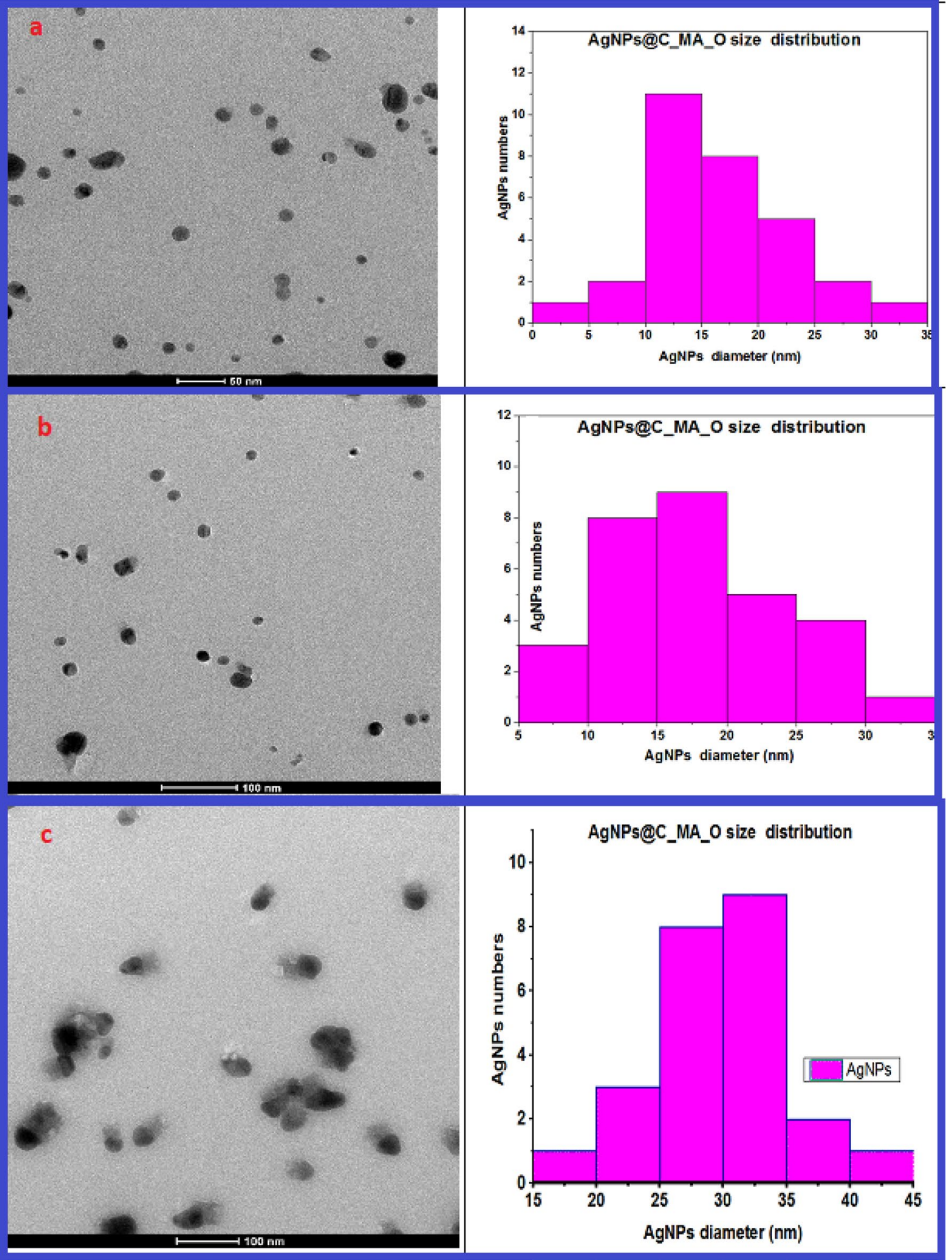
TEM images obtained for a suspended solution of AgNPs@C_MA_O represented in the left side at **(a)** at 50 nm and **(b,c)** at 100 nm magnification, respectively. The images **(a,b)** reveal that diameter AgNPs in range of (16 ± 6)nm and ( 18 ± 6) nm respectively. For stored over 6 months sample **(c)** average size of AgNPs was obtained approximately in range ( 27 ± 6) nm.

The TEM images (Fig. 5a–c, left sides) clearly show the formation of elongated spherical nanoparticles. A particle size histogram (Fig. 5 right sides) was obtained using ImageJ software. Figure 5a,b reveals that AgNPs@C_MA_O have a relatively homogenous distribution with the diameter in range (16 ± 6)nm and ( 18 ± 6)nm respectively. TEM image of the sample stored for over 6 months at room temperature (Fig. 5c) shows that the average size AgNPs shifted to higher values (27 ± 6)nm . The TEM and SEM images of AgNPs@C_MA_O confirmed that the AgNPs remained well-dispersed.

### Anticancer potential of AgNPs@C_MA_O

The development of effective therapies targeting cell death pathways is exceedingly complex owing to the number of therapeutic targets and varied toxicity profiles of single agents and combinations. However, it should be noted that the relationship between physical–chemical, including free surface energy and surface charge, covalent bonds, and crosslinking structures, and therapeutic effect is delicate. For instance, positively charged NPs are advantageous for trans-vascular transport, tumor penetration, and cellular uptake. It is generally explained as originating from the Coulombic attraction of the NP to the negatively charged cell membrane. On the other hand, high Zeta potential over 25 mV would increase cytotoxicity and impair colloidal stability ^34^^.^. Researchers also found that cellular uptake and toxicity are dependent on the size of the particles. Small particles tend to be more toxic owing to the ease of cellular penetration.

Here we present functionalized colloidal AgNPs (size between (16 ± 6) nm and (18 ± 6) nm; zeta potential + 18.2 mV) with ultra-stable dispersion in aqueous media. Carboxyl and amine containing corona of this AgNPs able to determine the vector of interaction in biological systems, control and cell uptake capabilities ^22,35,36^. We predict that wLEd approach and hydrogel compound combination, we can improve its anti-bacterial, anti-viral, and anti-cancer properties.

### Cytoxicity effects of AgNPs on cell viability

**Figure 6:**
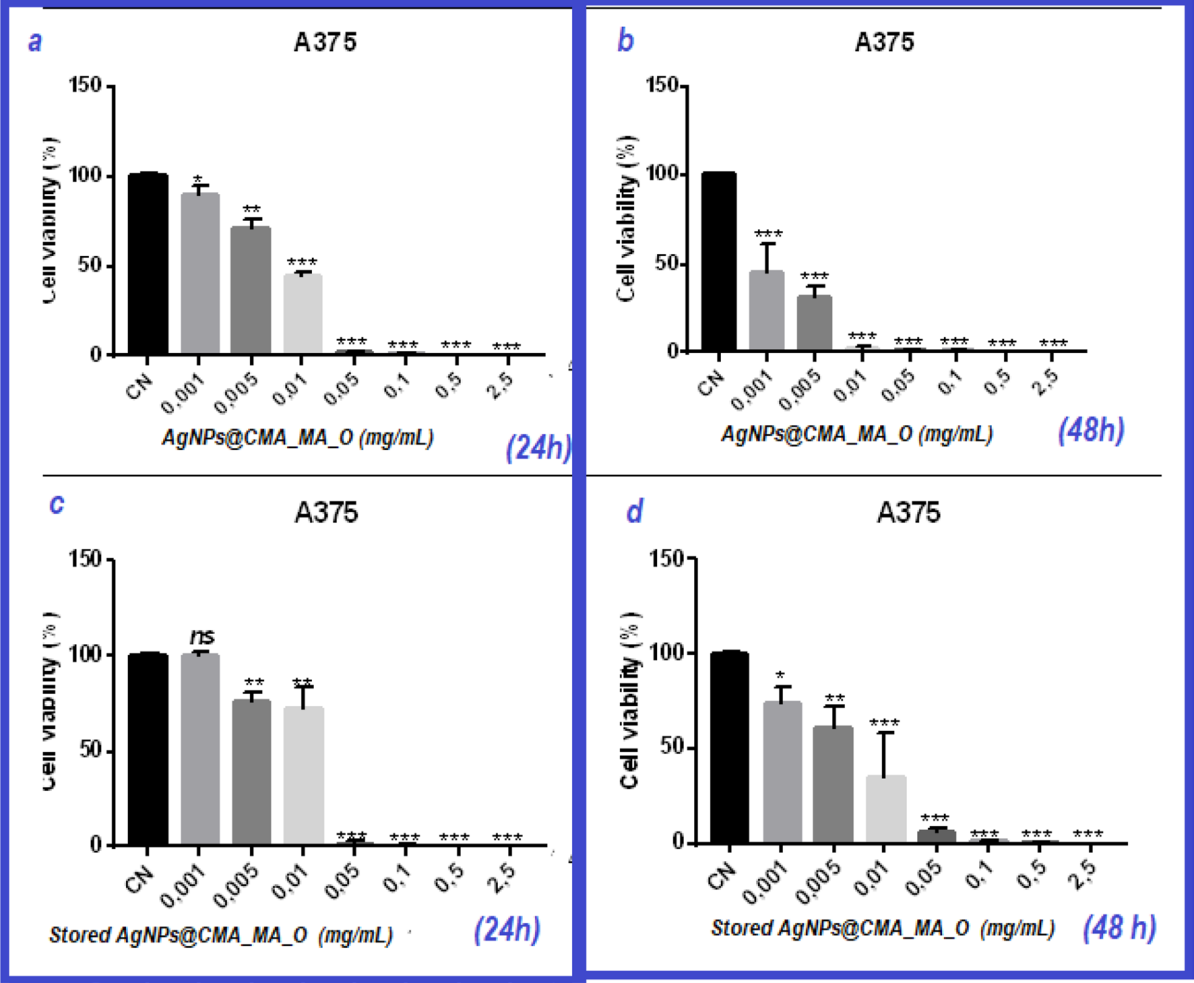
Cytotoxic effect of AgNPs@C_MA_O on melanoma A375 cancer cells. A375 cells were exposed to different concentrations of AgNPs : at 2.5, 0.5, 0.1, 0.05, 0.01, 0.005, 0.001 and 0 mg/ml concentrations for 24 h **(a)** and 48 h **(b)**; obtained results show reaching IC50 at 0.01 mg/mL and 0.005 mg/mL respectively. **(c,d)** demonstrates that after 6 months AgNPs did not lose their cytotoxic effect.

A MTT-based cell viability assay was applied to evaluate the effect of AgNPs@C_MA_O on melanoma A375 cells. The concentrations of AgNPs@C_MA_O hydrogel suspension were chosen based on our pilot study as well on our earlier study ^21^. In order to assess the cytotoxic effect of AgNPs@C_MA_O, human A375 melanoma cells were assessed for viability using the MTT assay after exposure to the following increasing concentrations of AgNPs for 24 and 48 h : at 0, 2.5, 0.5, 0.1, 0.05, 0.01, 0.005, and 0.001 mg/ml.. We found that AgNPs@C_MA_O displayed decreased viability of A375 melanoma cells in a dose- and time-dependent manner, reaching the half maximal inhibitory concentration (IC50) at 0.01 mg/mL and 0.005 mg/mL by 24 and 48 h time-point, respectively (Fig. 6a,b). Complete cell killing was achieved at 0.05 mg/ml. To evaluate the compound shelf life, we studied the cytotoxic effect of AgNPs@C_MA_O hydrogel stored for 6 months at room temperature (denoted as 'stored"), and found that the storage did not diminish the toxicity (Fig. 6c,d). Those results indicate that AgNPs@C_MA_O hydrogel has a low rate of aggregation, long shelf life and induce cancer cell cytotoxicity in a dose-dependent manner. In the present study, the IC_50_ of AgNPs was found as a 0.01 mg/mL, which is 33 times less than previously reported IC_50_ , as 0.33 mg/mL ^14^, 0.78 − 6.25 μg/mL and 12.5 mg/mL ^37^, 10.6 and 11.6 mg/mL ^38^ , 5 µg/mL ^39^ and 64.5 μg/mL ^40^ for different functionalized AgNPs.

This high percentage of cell death caused by a very low concentration of AgNPs could be due to the synergistic effect of AgNPs with its biocompatible hybrid hydrogel composition.

Chitosan and its functionalized derivatives and composites were reported ^14,41–44^ to possess potent biocompatibility, biodegradability and non-toxicity towards human fibroblast and to inhibit tumor growth by direct toxicity and immunomodulation ^45,46^. The presence of a functional group significantly inhibited the growth of tumor cancer cells whereas they do not show the cytotoxic effect on the healthy cells^46–50^. Furthermore, the cell viability of cells treated with 10, 20, 30, 40, and 50 μg/mL chitosan hydrogel coated AgNPs confirmed to show non-toxic effects via synergistic action to human fibroblasts ^51^ ; provided a significant efficiency against a large variety of microorganisms with minimal side effects ^52,53^, and exhibited good in vivo self-healing ability and biosafety^54^^.^ In this respect, it can be proposed that obtained AgNPs@C_MA_O Hydrogel not only prevents Ag NPs from premature interaction ^14,41,42,45,51–54^ with the biological environment but also helps in intracellular uptake and the induction of apoptosis ^21,43–50^^.^ Similar cell viability results at extra low concentrations with two different cell lines, where AgNPs was incorporated in a hybrid carboxylmethylcellulose base matrix, were archived in our previous study ^21^.

### Cell morphology

**Figure 7:**
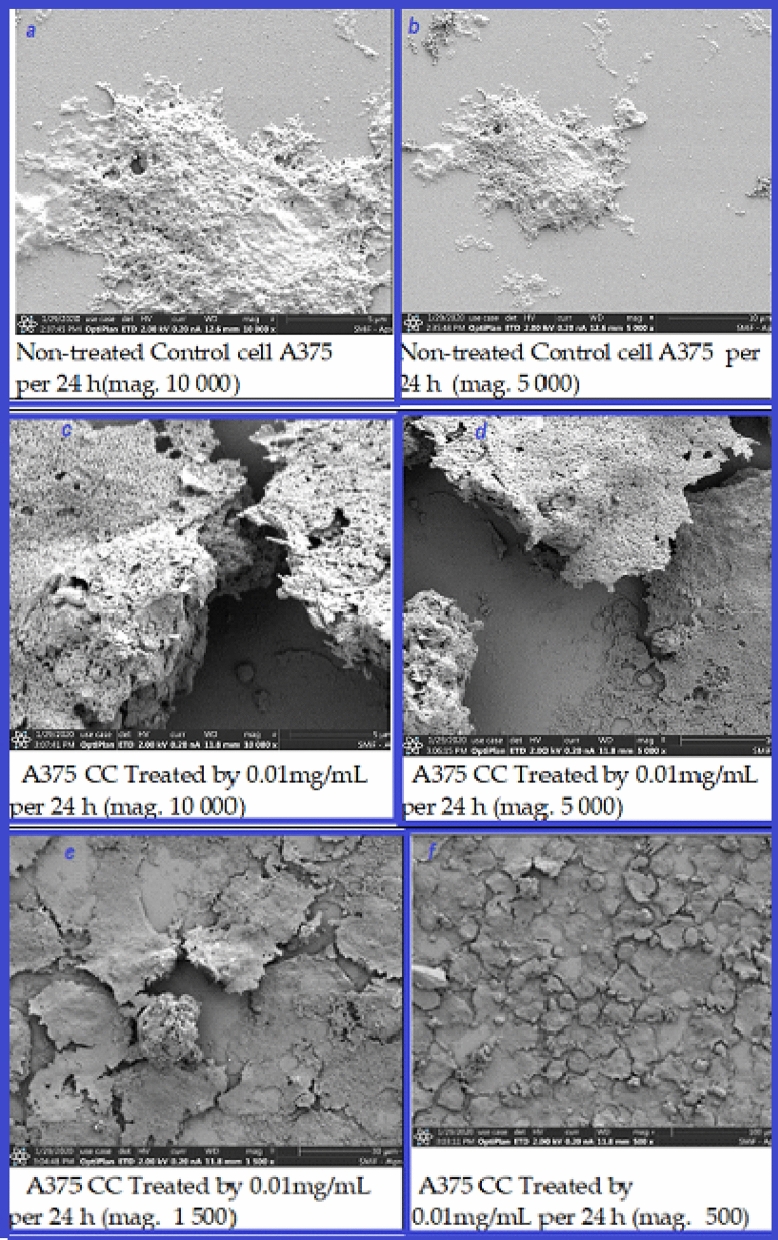
Representative SEM images of treated and non-treated A375 cancer cells. Untreated A375 cancer cells are represented in **(a,b)** at higher magnification—×10000 and ×5000, respectively. A375 cancer cells treated with 0.01 μg/mL of AgNPs@C_MA_O for 24 h are shown at **(c,d)** at higher magnification—10,000 and 5000; and 1500 and 500 magnification in **(e,f)**, respectively.

SEM was used to examine the appearance of A375 cells with AgNPs@C_MA_O at half-maximal inhibitory concentration (IC50) value (0.01 mg/mL). Representative SEM images of treated and non-treated A375 cancer cells are given at Fig. 7. As expected, untreated A375 controls cells which appeared with rounded surface and adherent to the tissue dishes (Fig. 7a,b). In contrast, AgNPs treated cells has marked morphological changes associated with apoptosis, membrane damage, detachment edges, and shrinkage (Fig. 7c–f) These studies indicated that AgNPs induced apoptosis or necrosis. We have also treated A375 cells with a decreased dose (0.005 mg/ml). SEM images (SI, Fig.S7) demonstrated that the 0.005 mg/ml dose did not show significant morphological changes and detachment, which is in accordance with the cell viability assay (Fig. 6a). These results indicate that the biocompatible hydrogel environment of the AgNPs plays a crucial role in cell death, which is translated by the morphological changes in the cells.

It is known that nanosilver often acts as a source of Ag ions inside the cell ^10,45,46^ which binds proteins and damages the cell membrane. The small size nanosilver particles can also enter into the cell through diffusion or endocytosis to cause mitochondrial dysfunction, generation of reactive oxygen species (ROS), leading to the damage of proteins and nucleic acids inside the cell. However, the mechanisms responsible for the apoptotic effect of AgNPs remain unknown. One major question we have is: what is the effect of surface coating on the toxicity of AgNPs and its relationship to the coexisting biomolecules. It is well known that attached functional groups change the physicochemical behavior of metal NPs and controls their targeting and anti-tumor effect. From a physicochemical point of view, the apoptotic mechanism is triggered by the combination of the hydrogen and solvation forces of Vander Waals and Coulomb interactions, which appear between functionalized AgNPs and the existing cellular environment. Based on this understanding, AgNPs@C_MA_O induces cell death through the following apoptosis pathways: (1) conjugation/complexing of AgNPs, the amine and carboxyl groups with similar groups of cancer cells , (2) the reaction of the carboxylic groups (COOH) with the amine groups of DNA and other reactive groups,which potentially contributed to the cancer cell growth, and (3) ODA with primary amino groups are capable of associating with similar reactive DNA macromolecules. Studies of AgNPs with the functional edges are available for molecular conjugation with the intracellular environment by having the virtue of relatively long-range (i.e. Coulomb) and as well as short-range (i.e. Vander Waals) forces.

The current study can be viewed as a first step understanding of the anti-tumor effect of these novel NPs. Future studies are required to decipher its role and determine its mechanism of action. It must be pointed out that the harmless wLEd irradiation as a soft approach synergized with the biocompatibility properties of the hybrid hydrogel during the synthesis exposure procedure.

Additionally, we used TEM to observe morphological and nucleic changes in AgNP-exposed cells. Due to COVID 2019, we did not have a chance to repeat these experiments, so we put obtained images as evidence in Supporting materials (SI, Fig.S8). Those TEM ımages illustrated that non -treated cells showed healthy cellular morphology, whereas treated cell images demonstrated the presence of Ag NPs inside the cells. A375 cells exposed to AgNPs2C_MA_O at 0.01 mg/mL concentration showed a distinct morphology, indicating necrosis.

## Conclusion

Our Novel wLEd approach was successfully used to obtain of light-driven functional novel soft material. Under wLED light, colloidal nanocomposite underwent a structural rearrangement where reactive complex linkages were easily transferred to the stable covalence forms. Recorded time dependent scattering spectral curves demonstrated that AgNPs were produced and reduced in-situ. We conclude that this harmless approach gave us a unique opportunity to maintain compatibility of AgNPs with biopolymer environments and improve anticancer potency obtained nanostructure. The resulting AgNPs@C_MA_O exhibited remarkable anti-cancer activities at very low dosages. Cell viability assay demonstrated that the concentration of novel functionalized AgNPs required to reduce the viability of A375 cells by 50% was 0.01 μg/mL, which is lower than previously reported. Moreover, at this concentration, AgNPs modified the morphology of the cell structure and increased cell death associated with disruptions in the cellular ultrastructure and cell membrane. These effects can be explained by the synergistic effect of AgNPs and the biocompatible hybrid hydrogel composition on the apoptosis pathway. The present findings of long-term stability of AgNPs@C_MA_O provide an added feature for a promising cancer therapeutic agent.

## Materials and methods

N-Carboxymethyl Chitosan, (Alaska snow crab shell) CAS Number: 83512-85-0 vg. molecular weight: 300 K Dalton was supplied by Qingdao Reach International Inc. Octadecylamine, 97% and Silver nitrate (AgNO3, 99.995%, melting point 202 °C with decomposition, d = 4.35 g cm^−3^) were purchased from Sigma-Aldrich.Poly(maleic acid-alt-acrylic acid) [poly(MAc-alt-AAc)] with content of maleic acid unit = 47.17 mass %, was purchased from Sigma-Aldrich (water solution with 50% concentration). Distilled water was used as a solvent. Human melanoma A375 cell line was obtained from the American Culture Collection (ATCC, Rockville, MD, USA).

### The IC50 protocol

The IC50 was obtained using GraphPAd Prism software. The is the half maximal inhibitory concentration (IC50) in order to measure of the potency of a substance in inhibiting a specific biological or biochemical function. IC50 is to plot x–y and fit the data with a straight line (linear regression). IC50 value is then estimated using the fitted line, i.e., Y = a * X + b, IC50 = (0.5—b)/a.

### Synthesis of the AgNPs@C_MA_O hydrogel

AgNPs in C_MA_O Hydrogel mixture was prepared by wLED mediated syntheses method at room temperature. Synthesis of novel AgNPs@C_MA_O was carried out by white led wLED -mediated syntheses method using the following procedures. Briefly, aqueous solution of Carboxymethyl Chitosan (3.3%) was added to the surfactant ODA (3.5 mass%) and mixed at room temperature for 3 h until formation of homogenous viscous liquid product: 0.8 mL of this solution was mixed with 0.2 mL poly(acrylic acid-co-maleic acid) and then AgNO3 precursor (salt, ~ 10 mass%) was added to this blend. After this the composition was stirred for 0.5 h until formation of homogenous blend and subjected to white LED irradiation treatments for 30–60 min. The obtained product was purified by following steps: after wLEd treatment, the AgNPs incorporated hydrogel was treated with a large amount of ethanol at room temperature by the intensive mixing up to the full precipitation of the product, which was isolated by centrifugation-filtration method and dried under the vacuum at 45-degree C.s.

### Characterization techniques

All syntheses, experiments and characterization (UV–Vis, DLS, FTIR, X-ray, SEM–EDX and TEM techniques) in this study were performed at the Shared Materials Instrumentation Facility (SMIF) DUKE University, N.C. SMIF consists of an Instrumentation Laboratory for the characterization and analysis of nanomaterials, and a Fabrication Laboratory and Cleanroom. Detailed information could be found here: https://smif.pratt.duke.edu/capabilities.

### Cell viability and calculation of cell viable percentage assay

Human melanoma A375 cell line cultured in 10% FBS/DMEM media (ThermoFisher Scientific) at 5% CO_2_ 37 °C incubator. Cells were plated in 96-well plates at a density of 2000 cells per well. Next day, cells were treated in triplicates with different concentrations of AgNPs. 24 h and 48 h later, cell viability was analyzed via colorimetric MTT (3-(4,5-dimethylthiazol-2-yl)-165 2,5-diphenyl tetrazolium bromide) assay. The absorbance was measured at 540 nm with using a microplate reader (MULTISKAN, Labsystems). Cell viability was normalized as percentages of untreated control cells.

### Treated SEM and TEM imaging protocol

Treated and non-treated cell samples for SEM and TEM imaging were prepared and analyzed with the assistance of the SMIF staff. A protocol for preparation of the treated cells is available in SI.

## Supplementary Information

Supplementary Information.
