# Supplementary material for: Novel light-driven functional AgNPs induce cancer death at extra low concentrations
Source: Sci Rep. 2021 Jun 24;11:13258. doi: 10.1038/s41598-021-92689-9 (PMC8225844; doi:10.1038/s41598-021-92689-9)
Supplement: Supplementary file 1 — Supplementary Information. [file 41598_2021_92689_MOESM1_ESM.docx]

Supporting information (SI)

TITLE: Novel light-driven functional AgNPs induce Cancer Death at extra low concentrations.

Authors: Ulviye Bunyatova ^1,2,*^ Manel Ben Hammouda ^3^; Jennifer Zhang ^3^

^1^ Baskent University, Biomedical Department, Engineering Facility, Ankara, Turkey,

^2^ Duke University, Department of Electrical and Computer engineering, Pratt School of Engineering Durham, North Carolina, USA ; *corresponding author; correspondence to [bunyatovau@yahoo.com]

^3^ Duke University, Department of Dermatology, School of Medicine, Durham, North Carolina, USA


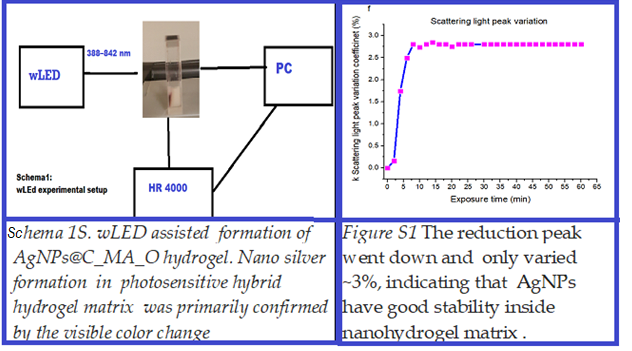


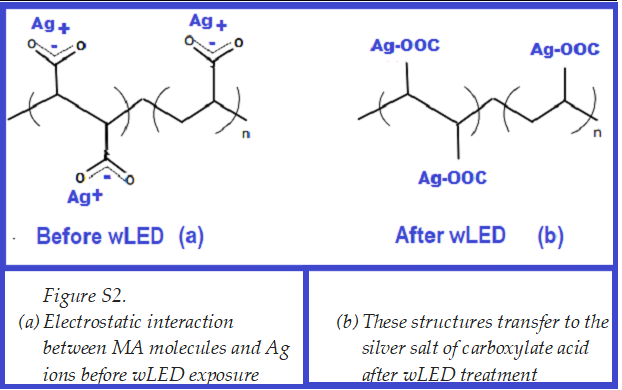


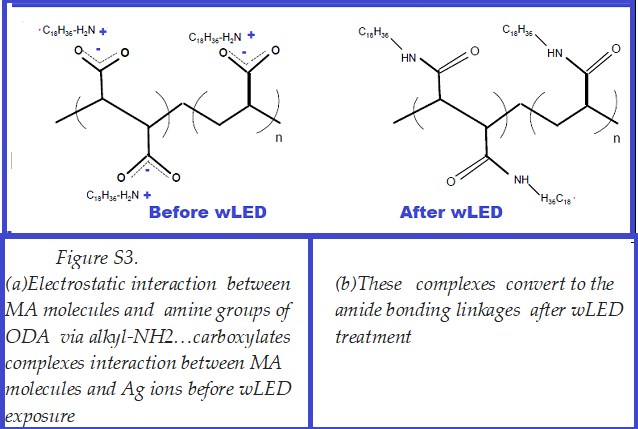


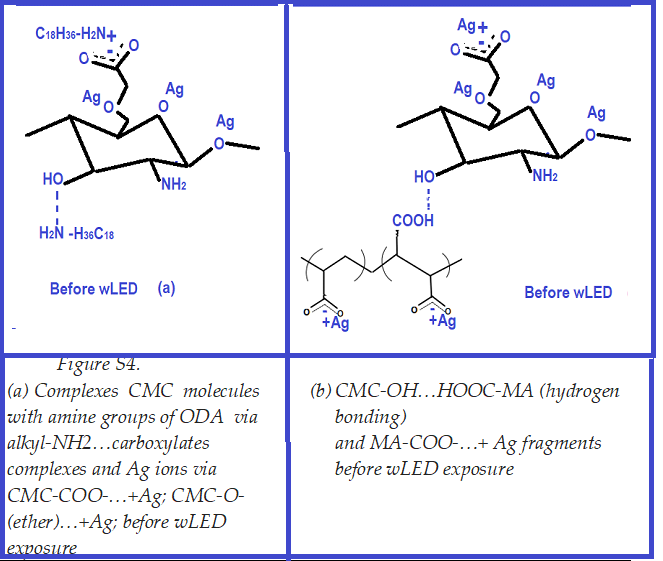

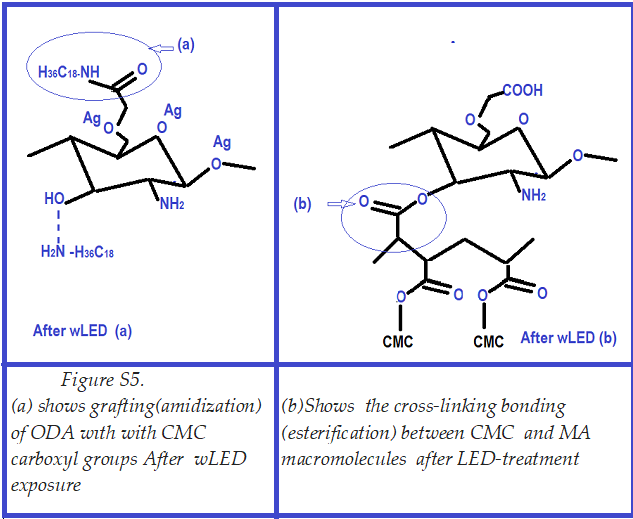


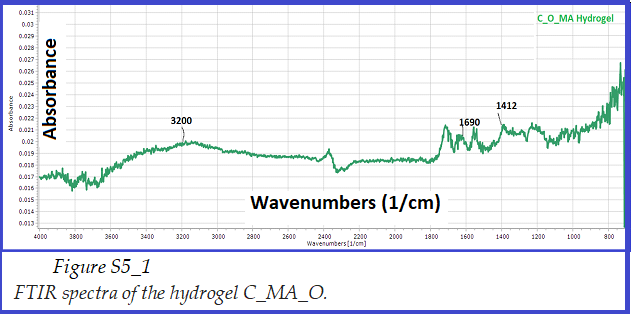


The broad peak in C_O_MA hydrogel at 3200 cm−1 is caused by both O–H and N–H stretching vibrations


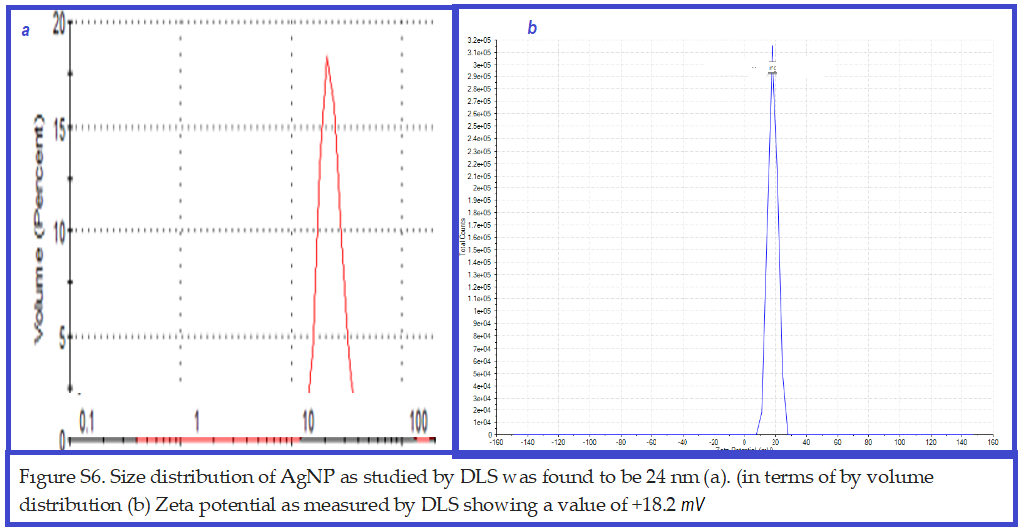


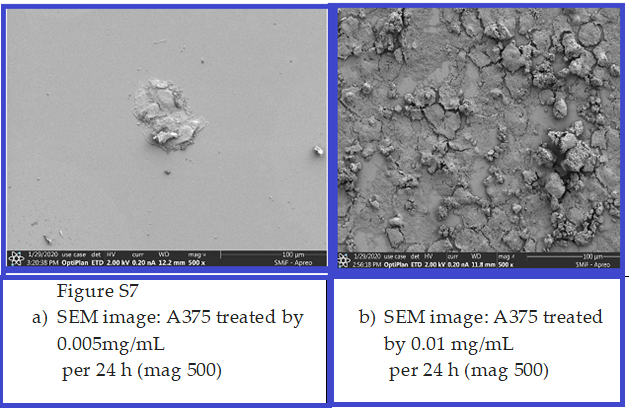


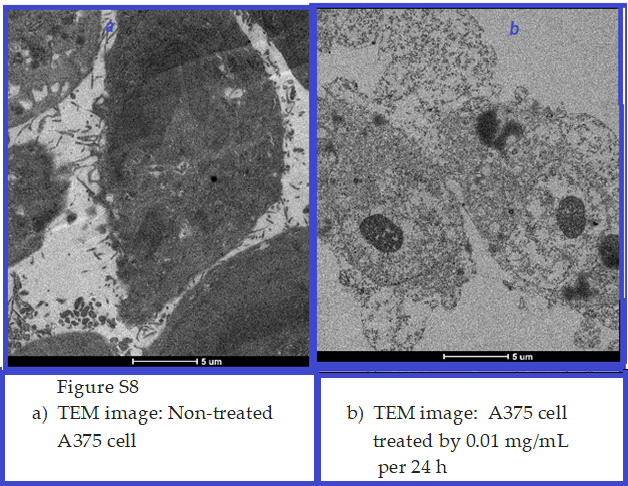


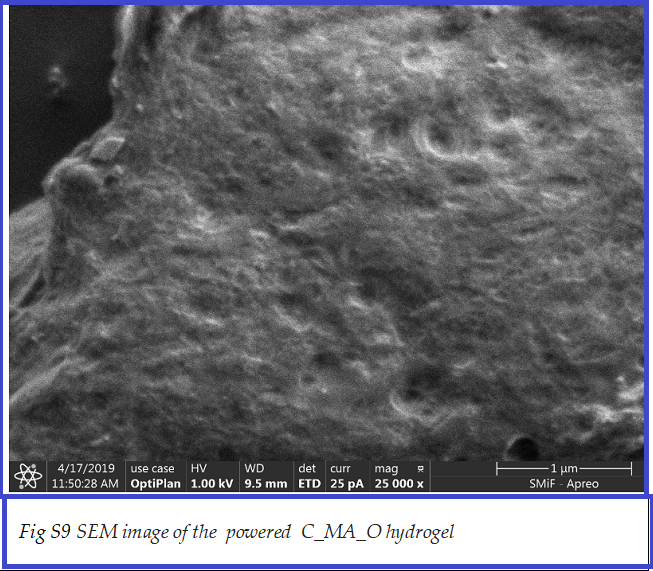


**Directions for Processing Biological SEM&TEM Samples**

1. Fixation of Samples:
2. Prepare fixative by combining the following ingredients (for 50ml fixative):
3. 10 mL 20% formaldehyde
4. 4 mL 25% glutaraldehyde
5. 5 mL 10X Phosphate buffered saline
6. 31 mL Distilled or other ultra pure water
7. Overlay fixative on the samples at room temperature

***Processing Samples (Day 1):***

**NOTE**: All rinse and stain volumes should cover the sample completely. At least 5mls for a 6 well plate.

1. Fixative is removed and the sample is washed twice with 1x PBS, at least 10 minutes each wash.
2. Remove PBS. 1% OsO_4_ is added to the sample to completely cover. The tube is sealed and covered (OsO_4_ is light sensitive). Stain for one hour.
3. Remove the OsO_4_ and rinse with 1x PBS, 2 times at least 10 minutes each time.
4. Remove 1x PBS and rinse with 0.1N acetate buffer, 1 time at least 10 minutes each time.
5. Remove acetate buffer and stain with 0.5% uranyl acetate (UA). Enough UA is added to completely cover the sample. The tube is sealed and covered. Stain for one hour.
6. Remove the uranyl acetate and rinse with 0.1N acetate buffer, 2 times at least 10 minutes each time.
7. After removing the last buffer rinse, wash twice, at least 10 minutes each time, with 30% ethanol.
8. Remove the 30% ethanol and wash twice, at least 10 minutes each time, with 50% ethanol.
9. Remove the 50 % ethanol and wash twice, at least 10 minutes each time, with 70% ethanol.
10. Remove the 70% ethanol and wash twice, at least 10 minutes each time, with 90% ethanol.
11. Remove the 90% ethanol and wash three times, at least 10 minutes each time, with 100% ethanol (200 proof).
12. Add the resin component, DDSA and NMA together in a plastic beaker (See attached sheet for making resin) and stir. Add the DMP-30 and continue to stir.
13. Remove the 100% ethanol and add resin. Allow the samples to sit overnight in the hood with the lids off.

***Embedding Samples (Day 2:)***

1. Make up new resin.
2. Place samples in the 50-60°C oven for 10 minutes (**with the lids off**).
3. Remove resin and replace with new resin. Let samples sit in the hood with the lids off for at least 10 mins.
4. Place samples in the 50-60°C oven for 10 minutes (with the lids off).
5. Replace resin with fresh resin, place labels in samples, place plates/dishes in oven and bake for 48 hours at 55-60°C.

# Preparation of Resin

Make the amount of resin required according to the following chart:

#### Component Units

####

Resin **grams** 53.3 42.6 40.0 32.0 26.7 21.3 13.3 10.7 5.35

DDSA **grams** 20.7 16.6 15.6 12.4 10.4 8.3 5.2 4.1 2.05

NMA **grams** 26.0 20.8 19.5 15.6 13.0 10.4 6.5 5.2 2.60

#### DMP-30 **mL** 1.4 1.12 1.05 0.84 0.70 0.56 0.35 0.28 0.14

## Total mL 100 80 75 60 50 40 25 20 10

***Volume***

***
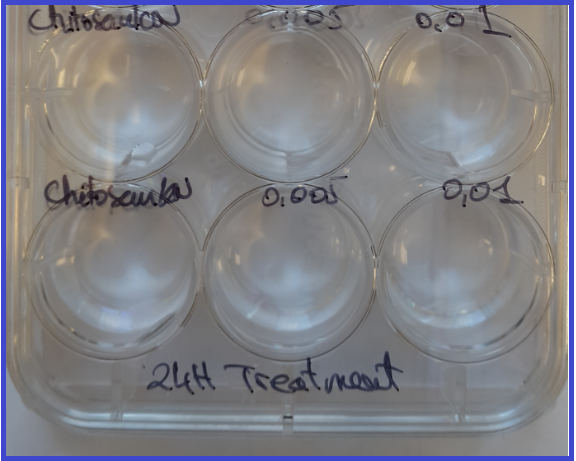
***
